# Potential Effect of Root Exudates from Ten Crops on Promoting Stress Tolerance in Alfalfa (*Medicago sativa*) Seedlings

**DOI:** 10.3390/life15040600

**Published:** 2025-04-04

**Authors:** Xiaoyan Zhang, Shangli Shi, Xiaolong Li, Changning Li, Qian Li

**Affiliations:** 1 College of Agriculture and Bioengineering, Longdong University, Qingyang 745000, China; lixiaol@st.gsau.edu.cn (X.L.); liqian08@lzu.edu.cn (Q.L.); 2 Key Laboratory of Grassland Ecosytem (Ministry of Education), College of Grassland Science, Lanzhou 730070, China; shishl@gsau.edu.cn (S.S.); licn@gsau.edu.cn (C.L.); 3 Centers for Grazing Land Ecosystem Sustainability, Gansu Agricultural University, Lanzhou 730070, China

**Keywords:** allelopathy, alfalfa, resistant physiology, root exudates, seed germination

## Abstract

Allelopathy plays a major role in agricultural production, influencing plant protection, crop yield, and crop rotation systems. This study investigated the effects of root exudates on 3105c alfalfa (*Medicago sativa*) seeds and seedlings to identify crops with strong and weak allelopathic potential. The results revealed that corn (*Zea mays* L.) (T1) exhibited the strongest allelopathic effects, whereas soybean (*Glycine max* (Linn.) Merr.) (T10) exhibited the weakest effects. T1 promoted seed germination by increasing radicle length and the simple vitality index. Both T1 and T10 promoted 3105c seedling growth and enhanced antioxidant capacity, albeit through different mechanisms. T1 primarily increased antioxidant capacity by elevating ascorbate and dehydroascorbate levels while reducing malondialdehyde content. In contrast, T10 enhanced antioxidant capacity by increasing soluble sugar and protein levels via hydroxyl free radical inhibition. These findings demonstrate that the allelopathic properties of corn effectively promote alfalfa growth by enhancing seed germination and improving physiological stress resistance.

## 1. Introduction

Alfalfa (*Medicago sativa*) is a legume grown across approximately 3.77 × 10^7^ km^2^ in China [1]. As one of the longest cultivated and most widely distributed leguminous forage species globally, alfalfa is rich in protein (approximately 20% of dry matter), minerals, vitamins, and carbohydrates. It plays a vital role in agricultural production and animal husbandry, providing high economic and ecological value [2]. However, unsustainable cultivation practices, such as continuous cropping, limit alfalfa production. The expansion of domestic animal husbandry is restricted by limited arable land, the need for environmentally friendly yield-increasing measures, and the regulation of alfalfa cultivation through natural chemistry.

In agricultural production, practices such as crop rotation, intercropping, weed control, and biological control exert allelopathic effects that influence the growth and development of neighboring plants [3]. Allelopathy refers to the direct or indirect effect of one plant on another through the release of chemical substances into the environment. These chemical substances are secondary metabolites. They play crucial roles in shaping plant interactions in natural and agricultural ecosystems [4]. Researchers have identified allelopathy between plants and applied it to agricultural production to reduce reliance on chemical pesticides and minimize their environmental impact [5,6]. The use of allelopathy to improve ecosystem and agricultural production sustainability offers new insights and approaches [7,8]. Cheema et al. applied allelopathic principles in crop management to suppress weeds and enhance plant growth in Pakistan, thereby improving crop productivity and environ-mental protection. They also synthesized a new type of environmentally friendly pesticide using allelochemicals [9].

Root exudates play a key role in the release and transport of allelochemicals, which mediate plant–plant interactions [10]. To maintain normal physiological metabolism, plants rely on antioxidant enzyme systems (enzymatic) and antioxidants (nonenzymatic) to eliminate reactive oxygen species (ROS) and maintain cellular homeostasis. Allelochemicals influence plants by modulating ROS production, redox balance, and antioxidant systems, including superoxide dismutase (SOD) [11] and peroxidase (POD) [12]. Numerous studies have reported that allelochemicals can inhibit antioxidant enzyme activity, leading to membrane lipid peroxidation and reducing the plant’s ability to remove ROS. Politycka et al. reported that vanillic acid can suppress POD and phenylalanine ammonia lyase activity [13]. Similarly, Li et al. reported that the phenolic acid of allelochemicals can increase cell membrane permeability, induce lipid peroxidation, and ultimately inhibit plant growth [7]. However, allelochemical effects vary among plants. Rama et al. showed that low concentrations of phenolic acid enhance plant defense enzyme systems by promoting SOD and POD activities [14]. Furthermore, Maqbool et al. found that the exogenous application of allelochemicals can increase endogenous receptor levels, thereby improving plant growth and resistance to abiotic stress [15]. Therefore, understanding the mechanisms by which allelopathy promotes or inhibits alfalfa growth and development is theoretically and practically important for its agricultural production.

In response to these problems, this study examined root exudates obtained from 10 common forage crops by inoculating them in alfalfa to assess their growth-promoting effects (strong and weak). Based on seed germination and seedling growth, two root exudates were selected. This study aimed to (a) provide a theoretical basis for root exudates that promote alfalfa growth, facilitating the establishment of a high-quality crop rotation system, and (b) clarify the different strategies of alfalfa in response to strong and weak allelopathy in the physiological aspects of stress resistance.

## 2. Materials and Methods

### 2.1. Plant Materials

In this study, *M. sativa* 3105c, which has a strong autotoxicity effect, was selected based on previous studies [16]. Seeds of 10 common crops (legume crops: red clover (Xuanli), white clover (Ruiwende), and soybean (Longdou No. 5); gramineous forage crops: maize (Longdan No. 338), wheat (Longzhong No. 10), wheatgrass (Nuodan), oats (Longyan No. 4), tall fescue (Aili), perennial ryegrass (Shandian), and smooth brome grass (Yuanye)) were provided by the Zhengdao seed Industry Co., Ltd., Beijing, China (Table 1).

### 2.2. Preparation of Root Exudates

Ten crops were cultivated using sand culture following our previous study [17]. First, a nutrient bowl filled with fine sand was placed in a square plastic basin (25.0 cm × 15.0 cm × 10.0 cm). High-quality seeds (two seeds for T1 and T10 and ten seeds for T2–T9) were rinsed with 0.1% HgCl_2_ solution, then evenly distributed in the culture bowl. The bowl was then placed in a light culture chamber (luminous flux density: 400 μmol m^−2^ s^−1^; 14 h light; 60% relative humidity; day and night temperatures: 25 ± 1 °C and 20 ± 1 °C, respectively). Seedlings were watered daily to ensure normal growth, and 100.0 mL of Hoagland nutrient solution was added daily for seven days [18].

Root exudates of 10 plants (T1–T10) were collected through water culture extraction. When T1–T10 seedlings (2 plants T1 and T10, 10 plants T2–T9) grew to about 8.0–15.0 cm in height, plant roots were carefully washed with distilled water to remove sand particles and surface impurities, and cleaned roots were placed in a plastic bowl (8 cm × 5 cm × 8 cm) with 300.0 mL of sterile distilled water in an artificial incubator for 72 h. The root exudate solution was collected and filtered with a 0.22-μm filter membrane to remove particulate matter, and the filtrates were drained in a freeze dryer. The concentration of the root exudate was determined based on the dry weight of the roots used. The final concentration of the leachate was 0.1 g∙mL^−1^ (dry root weight equivalent per milliliter of solution). The 10 kinds of root exudates were dissolved with an appropriate amount of deionized water. Root exudates were collected from independent biological replicates for each species. Specifically, exudates were collected from five individual plants of T2–T9 and two individual plants of T1 andT10, and each collection was processed separately to ensure biological replication. Finally, we obtained the root exudate (0.1 g∙mL^−1^) and these were stored at −20 °C.

### 2.3. Seed Germination and Seedling Growth Conditions

In stage 1, the alfalfa seeds were exposed to the ten crops root exudate. Alfalfa seeds were surface-sterilized and evenly distributed in separate Petri dishes (9.0 cm in diameter), with 30 seeds per dish. Each treatment was performed in quadruplicate. For treatments T1–T10, 3.5 mL of root exudates from 10 plant species was added to each Petri dish, whereas distilled water was used as a control (CK). The dishes were placed in an artificial climate box for germination (photoperiod 25 °C/12 h and a dark cycle 20 °C/12 h). On the seventh day, the number of germinated seeds was recorded, and then the simple vigor index (*SVI*), germination potential, and germination percentage were calculated. Additionally, the alfalfa seedling height, germ length, and radicle length were measured using Vernier calipers.

### 2.4. Calculation of Germination Parameters and Allelopathy Index

The germination percentage, germination potential, *SVI*, and allelopathy index (*RI*) were measured, as described in a previous study [19].

(1) $$Germination\;percentage=n_{7}/n_{total}\times 100\%$$

(2) $$Germination\;potential=n_{3}/n_{total}\times 100\%$$

(3) SVI=Germination percentage×Seeding height×100%

(4) RI=(T−C)/T (T≥C) or RI= (T−C)/C (T<C)

In Formulas (1)–(3), *n*_7_ indicates the number of germinated 3105c seeds after 7 days; *n*_3_ indicates the number of successful germinated seeds after 3 days; *n_total_* indicates the total number of seeds [20]. In Formula (4), *RI* represents the allelopathy index [19]; *C* represents CK; and *T* represents the treatment. The allelopathy comprehensive effect index (SE) was used to express the overall allelopathic effect. SE represents the arithmetic mean of the *RI* for the 10 plant root exudates across multiple test parameters of alfalfa seeds.

According to the seed germination analysis, T1 and T10 root exudates were selected for treatment of seedlings. These treatments determined the alfalfa seedling growth and physiological index responses to the T1 and T10 root exudates. Following previous research [17], the sand culture method was used to cultivate the 3105c alfalfa seedlings. On the 45th day, 0.1 g∙mL^−1^ of each root exudate was added to the nutrition bowls, whereas the same volume of distilled water was added to the CK group. Every other day, 100 mL of root exudate was added to maintain the solution concentration. To ensure uniformity, the solution was uniformly sprayed onto the sand surface in the seedling pots using a calibrated sprayer, and the pots were regularly rotated to minimize positional effects. After 21 days of continuous root exudate treatment, the alfalfa leaves were sampled and stored at −80 °C until further analysis.

### 2.5. Measurement of Physiological Indicators

To assess enzyme activities and ROS indicators, we conducted four biological replicates (independent plant samples) and three technical replicates (repeated measurements per sample) to ensure robustness and reproducibility. To minimize environmental variability and potential bias, we employed a randomized block design. To evaluate the effects of the root exudates on oxidative damage, the superoxide anion radical (O_2_^•−^), hydroxyl radical (OH^•^), hydrogen peroxide (H_2_O_2_), and malondialdehyde (MDA) indexes were measured. The MDA content was determined using the thiobarbituric acid method as described by Draper et al. [21]. The H_2_O_2_ content was measured using the KI colorimetric method [22]. The OH^•^ concentration was determined using the 2-deoxy-D-ribose colorimetric method [23]. The O_2_^•−^ production rate was determined using the p-aminobenzenesulfonic acid method, as described by Elstner et al. [24].

To determine the effects of the root exudates on the ROS scavenging system, antioxidant enzymes, including POD, SOD, APX, catalase (CAT), and glutathione reductase (GR), and antioxidants, including ascorbate (ASA) and dehydroascrobate (DHA), were selected. Alfalfa extraction was prepared by precooling and homogenizing 0.2 g of fresh leaves in ice-cold 50-mM phosphate-buffered saline (PBS) (0.1-mM EDTA, 1% PVP, pH 7.8). The homogenate was centrifuged at 12,000× *g* and 4 °C for 15 min. The supernatant was collected and stored in a refrigerator at −80 °C to determine the SOD, POD, and CAT activities. The activity of SOD was determined at 560 nm and was evaluated as the method described by Giannopolitis et al. [25]. The changes in POD activity absorbance value per unit time were measured at 470 nm and were evaluated as the method described by Chance et al. [26]. The changes in CAT activity absorbance value per unit time were measured at 240 nm and were evaluated as the method described by Havir et al. [27].

The APX activity was assessed as the method described by Murshed et al. [28]. The total reaction system included PBS (50-mM, pH 7.0), ASA (0.25-mM), 0.1-mM EDTA, 5-mM H_2_O_2_, and crude tissue extract. The changes in absorbance per unit time were determined at 290 nm. The activity of GR was assessed as the method described by Murshed et al. [28]. The total reaction system included PBS (50-mM, pH 8.0), EDTA (0.5-mM), NADPH (0.25-mM), GSSG (0.5-mM), and crude tissue extract. GSSG was added to initiate the reaction, and the changes in absorbance per unit time were determined at 340 nm. Preparation of alfalfa extracts by homogenization 0.5 g of leaf at 4 °C. The obtained homogenate was centrifuged at 22,000× *g* for 15 min to collect the supernatant for the determination of (ASA + DHA) and ASA. ASA and DHA were measured at 525 nm, as the method described by Murshed et al. [29].

To investigate the effects of root exudates on osmotic regulation, the indices of free proline (Pro), soluble sugar (SS), and soluble protein (SP) content were selected. The acid ninhydrin method was used to estimate the Pro content [30]. The anthrone colorimetry method was used to measure the SS content [31]. The bovine serum albumin was used to assess SP content based on a report by Bradford et al. [32]. The SP-756 P spectrophotometer (Shanghai spectrum Instrument Co., Ltd., Shanghai, China) was used for all colorimetric studies.

### 2.6. Statistical Analysis

Statistical analysis was carried out using R software (Version 4.0.2). (http://cran.rstudio.com/index.html (accessed on 14 February 2025)). The plyr package was used in TOPSIS analysis (technique for order of preference by similarity to ideal solution) to obtain the scores of 10 plants using the entropy-positive function to calculate the weights. The TOPSIS is a multi-index decision analysis and evaluation method. The Random Forest package was used in the Random Forest function to calculate and obtain the rank the relative importance of 3105c seed germination index. The differences in seed germination parameters and physiological parameters, between the treatment groups and the control group were compared using one-way ANOVA (significance level of *p* < 0.05). The avo function in the agricolae package was used for the calculations. Multiple comparison analyses were calculated using the duncan.test function in the agricolae package. The GgiraphExtra package in R was used for radar charts. The factoextra package was used in the principal component analysis (PCA). The principal component analysis (PCA) function in the PCAtools package was used for the calculations. The ggplot2 and ggpubr package was used to draw all figures.

## 3. Results

### 3.1. Effects of the Root Exudates from the 10 Plants on Seed Germination

**Figure 1:**
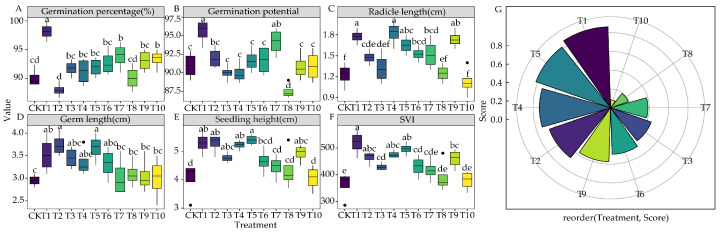
Effects of the root exudates from the 10 plants on alfalfa seed germination. (**A**) Germination percentage. (**B**) Germination potential. (**C**) Radicle length. (**D**) Germ length. (**E**) Seedling height. (**F**) Simple vigor index. (**G**) Comprehensive score ranking of the 10 plants. Ten colors represent the corresponding scores of T1–T10 root exudates. T1: corn; T2: wheat; T3: wheatgrass; T4: false oat grass; T5: red clover; T6: tall fescue; T7: white clover; T8: perennial ryegrass; T9: smooth brome grass; T10: soybean. The different lowercase letters indicate the significance in ten root exudates treatments.

The seed germination parameters of 3105c varied after treatment with different plant root exudates. Except for T2, all exudates promoted seed germination, with T1 exhibiting the most significant effect than others (Figure 1A) (*p* < 0.05). T1 and T7 in-creased the seed germination potential (*p* < 0.05), whereas T8 inhibited the seed germination potential (*p* < 0.05) (Figure 1B). Radicle length increased significantly under all treatments, except for T3, T8, and T10 (*p* < 0.05) (Figure 1C). Similarly, all treatments increased the germ length (Figure 1D). Regarding seedling height, all treatments, except for T7, T8, and T10, led to significant increases (Figure 1E). Moreover, the changing trend of *SVI* was similar to that of seedling length, with T1 exerting the most significant effect in promoting *SVI* (*p* < 0.05) (Figure 1F). We used TOPSIS to comprehensively analyze the seed germination parameters (germination percentage, germination potential, *SVI*, radicle length, seedling height, and germ length) across the 10 plant root exudates. Based on the corresponding scores of different treatments, T1 was identified as the optimal condition for promoting alfalfa 3105c seed germination, whereas T10 had no significant promoting effect (Figure 1G).

### 3.2. Allelopathy of the Root Exudates of the 10 Plants

**Figure 2:**
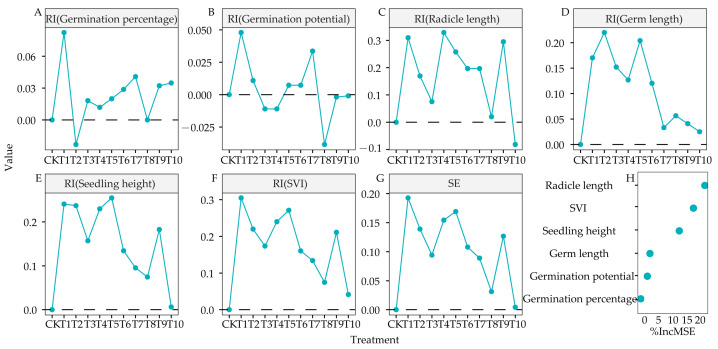
Effects of root extracts from 10 plants on comprehensive allelopathic effect. (**A**) Germiation percentage. (**B**) Germination potential. (**C**) Radicle length. (**D**) Germ length. (**E**) Seedling height. (**F**) Simple vigor index. (**G**) Allelopathy comprehensive effect index (SE) of alfalfa under 10 different root extracts. *RI* (allelopathy index) ≥ 0 indicates stimulatory effects, whereas *RI* ≤ 0 indicates inhibitory effects. (**H**) Relative importance of variables affecting SE in 3105c alfalfa.

The allelopathy index for germination percentage indicated that T2 inhibited seed germination, whereas other treatments had a promoting trend, with T1 showing the most pronounced promoting effect (Figure 2A). For germination potential, treatments T1, T2, T5, T6, and T7 exhibited promoting effects, whereas treatments T3, T4, T8, T9, and T10 exhibited inhibitory effects. Notably, T1 and T8 exerted the strongest promoting and inhibitory effects, respectively (Figure 2B). For radicle length, all treatments, except for T1 and T10, had stimulatory effects, with T5 exhibiting the most potent promoting effect (Figure 2C). Similarly, all treatments promoted germ length (Figure 2D) and seedling height, with T5 exerting the strongest promoting effect (Figure 2E). *SVI* increased under all treatments, with T1 exerting the strongest promoting effect (Figure 2F). Similarly, all treatments promoted the SE (SE > 0), with T1 exerting the strongest promoting effect (SE = 0.1926), whereas T10 exerted the weakest promoting effect (SE = 0.0041) (Figure 2G).

In this study, a Random Forest model was used to evaluate the variable importance of seed germination for SE. We determined the importance of relative variables in 3105c for different root exudates. The ranking of variable importance for seed germination parameters affecting SE was as follows: radicle length > *SVI* > seedling height > germ length > germination potential > germination percentage. Among these, radicle length was the most influential parameter, whereas germination percentage had the least effect (Figure 2H).

### 3.3. Effects of the Root Exudates on the Biomass and Height of Alfalfa Seedlings

**Figure 3:**
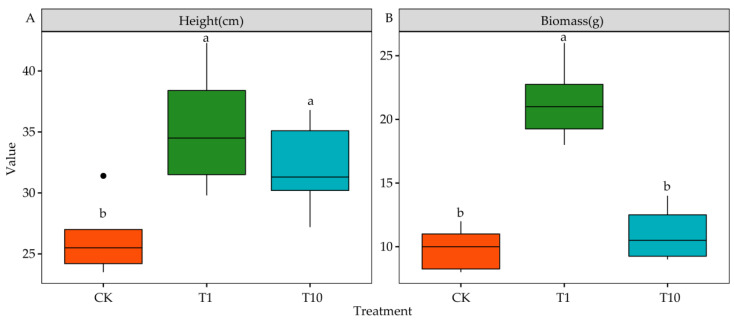
Effects of the root exudates on the biomass and height of seedlings. The orange, green, and blue indicate the CK, T1, and T10, respectively. T1 and T10 represent corn and soybean. The different lowercase letters indicate the significance in different treatments. (**A**) Height. (**B**) Biomass. Filled circles indicate outlier values.

The TOPSIS analysis identified T1 and T10 as having the strongest and weakest allelopathic effects, respectively. They both promoted plant height and biomass, albeit to different extents. Compared with CK, T1 and T10 significantly increased plant height by 34.12% and 22.04%, respectively (*p* < 0.05) (Figure 3A). Furthermore, compared with CK, T1 significantly increased biomass by 116.95% (*p* < 0.05), whereas T10 had no significant difference (*p* > 0.05) (Figure 3B).

### 3.4. Effects of Root Exudates on ROS Production and Lipid Peroxidation of Alfalfa Seedlings

**Figure 4:**
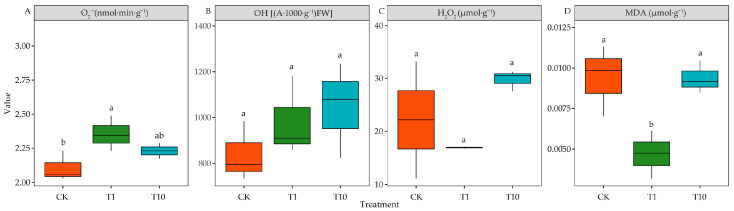
Effects of the root exudates on the lipid peroxidation of alfalfa. The orange, green, and blue indicate the CK, T1, and T10. T1 and T10 represent corn and soybean. The different lowercase letters indicate the significance in different treatments. (**A**) O_2_^•−^ content. (**B**) OH^•^ content. (**C**) H_2_O_2_ content. (**D**) MDA content.

Compared with CK, T1 significantly increased O_2_^•−^ levels (*p* < 0.05), but T10 showed no significant difference (*p* > 0.05) (Figure 4A). Both T1 and T10 had minimal effects on OH^•^ and H_2_O_2_ levels (Figure 4B,C) (*p* > 0.05). Interestingly, compared with CK, T1 significantly reduced MDA levels by 50.07% (*p* < 0.05), whereas T10 showed no significant difference (*p* > 0.05) (Figure 4D).

### 3.5. Effects of Root Exudates on Antioxidant of Alfalfa Seedling

**Figure 5:**
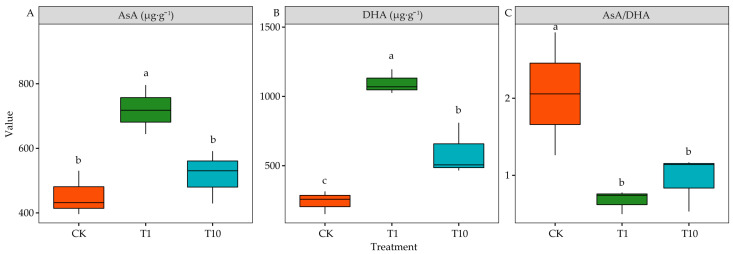
Effects of the root exudates on the antioxidant content. The orange, green, and blue indicate the CK, T1, and T10, respectively. T1 and T10 represent corn and soybean. The different lowercase letters indicate the significance in different treatments. (**A**) ASA. (**B**) DHA. (**C**) ASA/DHA.

Compared with CK, T1 significantly increased the ascorbic acid (ASA) content by 58.87% (*p* < 0.05), whereas it was not significant in T10 (*p* > 0.05) (Figure 5A). Compared with CK, T1 and T10 significantly increased the dehydroascorbic acid (DHA) content by 354.69% (*p* < 0.05) and 59.38% (*p* < 0.05), respectively. The DHA content in T1 was significantly higher than that in T10 (*p* < 0.05) (Figure 5B). Interestingly, both T1 and T10 significantly reduced the ASA/DHA ratio (*p* < 0.05), and there was no significant difference between T1 and T10 (*p* > 0.05) (Figure 5C).

### 3.6. Effects of the Root Exudates on the Antioxidant Enzyme Activities of Alfalfa Seedlings

**Figure 6:**
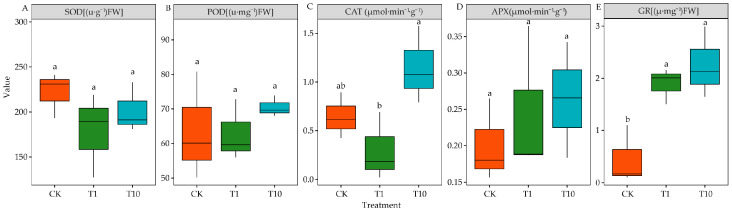
Effects of the root exudates on the antioxidant enzyme activities. The orange, green, and blue indicate the CK, T1, and T10, respectively. T1 and T10 represent corn and soybean. The different lowercase letters indicate the significance in different treatments. (**A**) SOD. (**B**) POD. (**C**) CAT. (**D**) APX. (**E**) GR.

Compared with those in the CK, the SOD, POD, and APX activities in T1 and T10 were not significantly different (*p* > 0.05) (Figure 6A,B,D). However, T10 significantly increased the CAT activity by 78.72% (*p* < 0.05), whereas T1 had no significant effect (*p* > 0.05) (Figure 6C). It is worth noting that the GR activity was significantly increased by 314.62% and 394.62% in T1 and T10, respectively (*p* < 0.05) (Figure 6E).

### 3.7. Effects of the Root Exudates on the Osmotic Adjustment Substance of Alfalfa Seedlings

**Figure 7:**
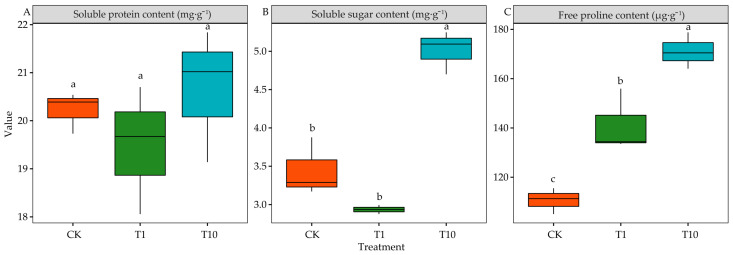
Effects of the root exudates on the osmotic adjustment substance. The orange, green, and blue indicate the CK, T1, and T10, respectively. T1 and T10 represent corn and soybean. The different lowercase letters indicate the significance in different treatments. (**A**) SP content. (**B**) Soluble sugar content. (**C**) Free proline content.

Compared with CK, the SP in T1 and T10 was not significantly different (*p* > 0.05) (Figure 7A). T10 significantly increased the SS by 45.53% (*p* < 0.05), whereas T1 had no significant effect (*p* > 0.05) (Figure 7B). Additionally, the Pro in T1 and T10 was increased by 27.69% and 54.63% compared with CK, respectively (*p* < 0.05). Moreover, the Pro was significantly higher in T10 than in T1 (*p* < 0.05) (Figure 7C).

### 3.8. Responses of the Root Exudates in Promoting the Resistant Physiology of Alfalfa

**Figure 8:**
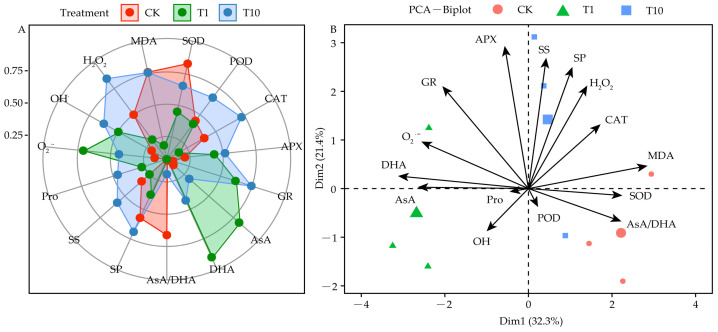
Changes in various physiological parameters and principal component analysis (PCA). (**A**) Effects of the root exudates on the physiological parameters of alfalfa, as visualized in radar charts. (**B**) PCA of the physiological parameters. The orange, green, and blue represent the CK, T1, and T10, respectively. T1 and T10 represent corn and soybean.

The radar chart in Figure 8A illustrates the effects of T1 and T10 on ROS production (O_2_^•−^, OH^•^, and H_2_O_2_) and lipid peroxidation parameters (MDA). T1 increased O_2_^•−^ and OH^•^ levels but decreased H_2_O_2_ and MDA levels. Interestingly, T10 increased the OH^•^, O_2_^•−^, and H_2_O_2_ levels but did not reduce MDA levels compared with CK. For antioxidant indicators, T1 increased APX and GR activities, and ASA and DHA, content but reduced SOD and CAT activities and the ASA/DHA ratio. Conversely, T10 increased POD, CAT, APX, and GR activities as well as ASA and DHA levels, but reduced SOD activity and the ASA/DHA ratio. T1 significantly increased the ASA content compared with T10. For osmotic adjustment substances with a positive effect, T1 increased the Pro levels and reduced the SS and SP levels, whereas T10 increased the Pro, SS, and SP levels (Figure 8A).

The PCA of the stress resistance indicators (Figure 8B) showed that CK, T1, and T10 formed three different groups. Dimensions (Dim.) 1 and 2 accounted for 53.7% (32.3% + 21.4%) of the total information contained. Additionally, the distances and angles between the geometric centers of each treatment and the indexes revealed that CK increased the ASA/DHA ratio but inhibited O_2_^•−^ accumulation. T1 promoted ASA and DHA accumulation and inhibited MDA accumulation. Meanwhile, T10 promoted SS and SP accumulation and inhibited OH^•^ accumulation.

## 4. Discussion

### 4.1. Strong and Weak Allelopathy and Its Effects on the Growth of Alfalfa

Allelopathy between plants is a natural phenomenon in chemical ecology [4]. When two plants affect each other, there are complementary positive effects, such as cultivation management and weed control, and adverse effects that restrict each other, such as autotoxicity and biological invasion. Root exudates are essential to produce allelopathy between plants [33]. It is a vital carrier in exchanging and transmitting information between plants and soil, and it is also the primary regulator of rhizosphere dialogue between plants [34]. This study found that T1, T2, T3, T4, T5, T6, T7, T8, T9, and T10 had uneven effects on the seed germination parameters of alfalfa 3105c. However, compared with the CK, T1 had the best performance in all germination parameters (Figure 1A–F), as its root exudates could promote or inhibit the growth of other plants and directly affect the germination rate, germination index, germination vigor, and radicle length [20]. Furthermore, the TOPSIS analysis showed that corn root exudates (T1) and soybean root exudates (T10) scored the highest and lowest, respectively (Figure 1G). Similarly, according to the analysis of *RI* proposed by Williamson et al. [20]. T1 performed the best in promoting all the germination parameters of alfalfa under the 10 plants root exudates (Figure 2A–F). The SE of 3105c seeds under the 10 plants root exudates treatments was also different. T1 performed outstandingly in promoting seed germination, whereas T10 had the least promoting effects (Figure 2G). Previous studies have isolated substances that stimulate sunflower seed germination in corn root exudates [35]. Additionally, according to the variable importance evaluation function of the random forest model, radicle length and *SVI* were the main parameters of SE in different treatments (Figure 2H). We speculated that the allelochemicals that stimulate the seed germination of alfalfa exist in corn root exudates. T10 showed the most minor effect on 3105c seed germination, which might be due to the allelochemicals in soybean root exudates. Further, we mainly focused on studying the effects of T1 and T10 on 3105c seedlings.

Allelochemicals can stimulate or inhibit seed germination and plant growth [36]. Inal et al. reported that the intercropping of corn with peanut and medicinal materials with peanut can achieve a certain yield increase effect in some areas [37]. Numerous studies have shown that root exudates have varying degrees of influence on other plant growth and development. This study found that both T1 and T10 increased the height of 3105c seedlings, but only T1 increased its biomass. Dai et al. (2009) also reported the intercropping of peanuts with Euphorbia japonicus and Atractylodes lanceolata increases the per plant yield by >30% [38]. Shen et al. reported that an increase in plant root exudates may respond to nutrient requirements [39]. It can also promote plant growth and development by directly or indirectly adjusting the biological and non-biological factors in the soil. The significant differences in the root exudates affected the growth and development of 3105c due to the different composition or content of allelochemicals released by corn and soybean. Moreover, 3105c seedlings were not highly sensitive to T10, while T1 promoted its growth and development due to certain allelochemicals in corn root exudates. Guo et al. found that corn root exudates can indirectly promote plant growth and development by repairing organic pollutant soil, improving microbial activity and diversity, and controlling soil-borne diseases [40]. Scheffknecht et al. believed that corn root exudates provided the necessary carbon and nitrogen sources for the survival of certain microorganisms in the soil, these also increased the number and activity of soil microorganisms [41]. Furthermore, Niemeyer et al. found that 2-hydroxy-2H-1, 4-benzoxazin-3(4H)-one is the most abundant bacteriostatic substance in the root system secreted into the rhizosphere by corn [42]. It can inhibit the release, swimming, spore germination, and hyphal growth of Phytophthora nicotianae zoospores [43]. The studies above showed that corn root exudates contain allelochemicals that directly or indirectly promote plant growth and development. However, its specific components and mechanism of action need to be further explored.

### 4.2. Alteration of Oxidative Damage in Alfalfa Under Strong and Weak Allelopathy

ROS accumulation and membrane lipid peroxidation are essential characteristics of plants suffering from abiotic and biotic stress. ROS production and removal, and redox balance in cells, play a vital role in allelopathy [44]. This study found that T1 increased the O_2_^•−^ content of alfalfa 3105c, while both T1 and T10 had no significant effects on H_2_O_2_ and OH^•^. Plants produce much ROS, such as O_2_^•−^, H_2_O_2_, and OH^•^, after being exposed to allelochemicals [45]. Large quantities of accumulated ROS can cause cell membrane damage, destroying plant photosynthesis and respiration, causing plant death in severe cases [46]. Abiotic and biotic stress can cause plant damage, which has a close relationship to membrane peroxidation caused by ROS accumulation. T1 significantly reduced the MDA content compared with CK and T10. MDA is an essential product of membrane lipid peroxidation. As a result, the MDA content represented the degree of membrane lipid peroxidation [47]. This indicated that the two treatments did not cause severe oxidative damage to 3105c compared with CK, and T1 significantly reduced the membrane lipid peroxidation. T1 and T10 had a little effect on ROS, and the ROS produced was within the controllable range. The plant can eliminate ROS accumulation by adjusting its antioxidant enzyme activity or antioxidant content. T1 can also increase the defense enzyme activity and antioxidant content of 3105c and reduce membrane lipid peroxidation. Hu et al. also found that corn roots can release benzoxazine, promoting the release of jasmonic acid signal substances and improving their defense capabilities [48].

### 4.3. Alteration of the Antioxidant System and Osmotic Adjustment Substance in Alfalfa Under Strong and Weak Allelopathy

The ASA-GSH pathway plays a vital role in oxidative stress induced by abiotic stress [49]. In higher plants, ASA is one of the most abundant soluble antioxidants, and it plays a vital role in direct ROS elimination by participating in the ASA-GSH cycle [50]. This study found that T1 significantly increased the ASA compared with the CK and T10. Both T1 and T10 significantly increased the DHA content, and it significantly reduced the ratio of ASA to DHA. This indicated that T1 can significantly promote ASA accumulation and increase the antioxidant capacity of 3105c. This can explain why the MDA content in T1 was significantly lower than that in CK and T10 (Figure 4D). However, T1 and T10 significantly increased the DHA content and significantly reduced the ratio of ASA to DHA. The two treatments did not cause oxidative damage (Figure 4A–C). T1 increased the ASA content of 3105c, improving its ability to maintain a redox balance and enhancing its antioxidant capacity. At the same time, T10 had no promoting effect on the antioxidant system. Terzi et al. reported that mitigating the oxidative stress mediated by ASA can enhance the defense mechanism of plant antioxidants. Increasing the endogenous ASA content can effectively reduce H_2_O_2_ production and alleviate membrane lipid peroxidation [51].

This study found that SOD, POD, and APX activities were not significantly different in the two treatments. However, T10 significantly increased the CAT activity, whereas both T1 and T10 significantly increased the GR activity. The results showed that the two treatments did not stimulate the SOD, POD, and APX activities. Some studies found that plants can resist oxidative damage by increasing the non-enzymatic antioxidant content and antioxidant enzyme activity [52]. SOD, POD, and CAT are crucial antioxidant enzyme systems that can eliminate ROS in plants under abiotic stress. SOD is the first enzyme to fight against oxidative damage mediated by reactive oxygen free radicals [53]. It is a key enzyme in the protective enzyme system—it removes excess superoxide anions in cells, disproportionate O_2_^•−^ to H_2_O_2_, and eliminates the toxicity of superoxide anions [54]. T1 and T10 had a little effect on ROS (Figure 4A–C). Therefore, the antioxidant capacity of SOD, POD, and APX was not activated. In addition, POD and CAT have a decomposing effect on H_2_O_2_ [55]. Similarly, H_2_O_2_ is finally converted to H_2_O by APX catalysis in the ASA-GSH metabolic pathway [56]. Some studies reported that GR also plays a vital role in ASA regeneration [57]. GR plays an important role in regulating abiotic stresses. GR gene overexpression can enhance the tolerance of plants to heavy metals, salt, and drought stress [58,59]. T1 and T10 significantly increased the GR activity, but T10 did not increase the ASA content, possibly because T1 promoted the ASA-GSH cycle and increased the ASA content by up-regulating the GR activity and enhancing the resistance of 3105c. T10 improved the antioxidant capacity by increasing the CAT activity of 3105c, so it was different in improving the antioxidant capacity of 3105c in T1.

The osmotic regulation of plant cells is one of the major ways to adjust the environment and resist abiotic stress. Plants achieve osmotic adjustment by producing a large number of ions and compatible solutes (Pro, SS, SP, organic acids, glycine betaine, etc.) [60]. This study found that the SS content was significantly increased in T10, whereas the SP content was not significantly changed in the two treatments. T1 and T10 could significantly increase the Pro content of 3105c. This showed that the two treatments promoted the osmotic adjustment ability of 3105c, thereby increasing its stress resistance ability. Pro can promote water absorption and ROS quenching, thereby improving plant resistance and protecting tissues away from damage [61]. Molinari et al. also found that plants under drought stress can protect their photosynthetic system from peroxide damage by increasing the Pro [62].

### 4.4. Difference of Physiological Stress Resistance Pathway in Alfalfa Between Strong and Weak Allelopathy

ASA is positively correlated with plant stress resistance. Increasing the ASA content or promoting the reduction of DHA levels can improve stress resistance [63]. The PCA showed that T1 mainly increased the antioxidant capacity and growth of 3105c by increasing the ASA and DHA content and by reducing the MDA content. T10 mainly affected the antioxidant capacity, growth, and development by increasing the SS and SP levels. On the contrary, T10 decreased the OH^•^ content (Figure 8B). The two treatments inspired two completely different promotion strategies. However, according to the plant height and biomass analysis of 3105c under T1 and T10, T1 resulted in a better growth and development (Figure 3A,B). We speculate that T1 stimulate 3105c seedlings to increase the ASA and DHA content and reduce the MDA content. T1 might improve its antioxidant capacity, growth, and development. ASA can increase the antioxidant capacity during plant growth and development, it can also affect plant carbon metabolism, cell division, growth, stress response, ion absorption, and other physiological functions [64]. Moreover, ASA can also interact with DNA demethylation [65].

## 5. Conclusions

In this study, root exudates from 10 different plants enhanced alfalfa seed germination, with corn root exudates, particularly stimulating the germination process. Both corn and soybean root exudates promoted the growth and antioxidant capacity of alfalfa seedlings, although the effects differed due to variations in allelochemical composition. Notably, corn root exudates had a more pronounced growth-promoting effect than soybean root exudates. Additionally, these exudates influenced alfalfa’s physiological stress resistance through strong and weak allelopathy. Corn root exudates enhanced antioxidant capacity by increasing ASA and DHA content while reducing MDA content. Soybean root exudates increased SS and SP levels while inhibiting OH^•^ accumulation. However, this study only investigated the effects of different forage root exudates on alfalfa seeds and seedlings. However, this study only represents the effects of different kinds of forage root exudates on alfalfa seeds and seedlings under laboratory conditions. Further long-term research is needed to comprehensively evaluate the allelopathic effects of different crops under field conditions.

## Figures and Tables

**Table 1:** Variety name and serial number of plants.

Number	Varieties	Latin Name
T1	Maize	*Zea mays* L.
T2	Wheat	*Triticum aestivum* L.
T3	Wheatgrass	*Agropyron cristatum* L. Gaertn
T4	Oats	*Avena sativa* L.
T5	Red clover	*Trifolium pratense* L.
T6	Tall Fescue	*Festuca elata* Keng ex E. Alexeev
T7	White clover	*Trrifolium repens* L.
T8	Perennial ryegrass	*Lolium perenne* L.
T9	Smooth brome grass	*Bromus inermis* Leyss.
T10	Soybean	*Glycine max* (Linn.) Merr.

## Data Availability

The data presented in this study are available on request from the corresponding author.

## Abbreviations

APX, ascorbate peroxidase; ASA, antioxidants, ascorbate; ASA/DHA, ratio of reduced to oxidized ascorbate; ASA-GSH, ascorbate-glutathione; CAT, catalase; DHA, dehydroascrobate (oxidized ascorbate); EDTA, ethylenediamine tetraacetic acid; GR, glutathione reductase; GSSG, oxidized glutathione; H_2_O_2_, hydrogen peroxide; MDA, malondialdehyde; NADPH, nicotinamide adenine dinucleotide phosphate; O^2•−^, superoxide anion free radical; OH^•^, hydroxyl free radical; PAL, phenylalanine ammonia lyase; PBS, phosphate-buffered saline; PCA, principal component analysis; POD, peroxidase; PVP, polyvinylpyrrolidone; *RI*, response index; ROS, reactive oxygen species; SE, strongest promoting effect; SOD, superoxide dismutase; SP, soluble protein; *SVI*, simple vigor index; TOPSIS, technique for order of preference by similarity to ideal solution.
